# Differences of Lipid Profile Among Ischemic and Hemorrhagic Stroke Patients in a Tertiary Hospital in Riyadh, Saudi Arabia: A Retrospective Cohort Study

**DOI:** 10.7759/cureus.25540

**Published:** 2022-05-31

**Authors:** Hadeel Alkhaneen, Demma Alsadoun, Leen Almojel, Alhanoof Alotaibi, Amal Akkam

**Affiliations:** 1 Family Medicine Department, King Abdulaziz Medical City, Riyadh, SAU

**Keywords:** dyslipidemia, hemorrhagic stroke, ischemic stroke, kingdom of saudi arabia (ksa), hypertension

## Abstract

Objective

This study aims to compare the serum lipid profiles of patients with ischaemic and hemorrhagic strokes.

Study design

This was a retrospective, comparative study.

Place and duration of the study

The study was conducted at Military Hospital, Riyadh, from January 1, 2018, to December 31, 2020.

Methodology

Patients with a diagnosis of stroke who presented to the emergency department and was confirmed to have ischemic or hemorrhagic strokes by computed tomography (CT) from January 1, 2018, to December 31, 2020. This study was based on data extracted from an electronic hospital information system (BESTCare) of patients presenting to King Abdulaziz Medical City, National Guard Health Affairs, which is a tertiary medical center in Riyadh, Saudi Arabia. Patients who lack lipid profile reading within six months before the incident or had a traumatic hemorrhagic stroke were excluded from the study. All these variables were included in the study: age, gender, height, weight, date of the incident, date of last lipid profile results, type of stroke, comorbidities, on a statin or not, and lipid profile including (high-density lipoprotein cholesterol (HDL-C), low-density lipoprotein cholesterol (LDL-C), total cholesterol, and triglycerides). Microsoft Excel 2019 (Microsoft Corporation, Redmond, WA) was used for data entry and data cleaning, and Statistical Package for the Social Sciences (SPSS) version 23 (IBM Corp., Armonk, NY) was used for data analysis and visualization of the results.

Results

The mean age of presentation of stroke was 68±13, 59% of patients were males, and 41% were females. BMI ranged from 30±8. Obesity (BMI 30 or above) was predominant in both stroke subtypes. Among all patient comorbidities, hypertension was the most predominant. Diabetes was present in 71% of the population. Of the participants in this study, 114 had ischemic stroke and 87 had a hemorrhagic type. A comparison of the serum lipid profile of two categories of strokes showed no statistical significance in serum values of total cholesterol, triglycerides, and LDL-C in ischemic and hemorrhagic stroke patients.

## Introduction

A stroke or cerebrovascular accident (CVA) is the most common neurological cause of morbidity and mortality all over the world. It is the second leading cause of death worldwide, accounting for 165,000 deaths occur each year in the United States alone [1]. According to the World Health Organization (WHO), stroke was the second largest cause of death in Saudi Arabia, being accountable for 14,400 deaths in 2012 [2]. It is the leading cause of adult functional disability, with 15%-30% of survivors being permanently disabled [2].

It involves the rapid loss of brain function caused by a disruption of blood supply to the brain triggered by ischemia (lack of blood flow), blockage (thrombosis, arterial embolism), or hemorrhage [3-4]. The prevalence of stroke in the Middle East ranged between 508 and 777 per 100,000 population [3]. According to a recent study, the incidence of stroke is 29 stroke cases for every 100,000 people annually for individuals residing in Saudi Arabia [4]. There are various risk factors, such as age, gender, family history, and other modifiable risk factors like lifestyle habits such as tobacco, alcohol use, sedentary life, obesity, high blood pressure, hyperlipidemia, diabetes, and atherosclerosis [1]. Furthermore, serum lipid level has an established effect on short-term mortality in older patients with stroke, thus it should be considered the main target for primary and secondary prevention of stroke [5]. In literature, the relationship between lipid profile and subtypes of stroke remains unclear [1].

Several studies demonstrated an inverse relationship between total cholesterol and the prevalence of hemorrhagic stroke [6]. On the other hand, other clinical studies showed ﻿an increased risk of ischemic stroke rates at higher levels of total cholesterol [2]. ﻿Triglycerides showed to increase the risk of both ischemic and hemorrhagic strokes [6-7]. ﻿Lower HDL-cholesterol levels were found to be significantly higher in ischemic stroke patients compared to hemorrhagic stroke [1].

This study aimed to evaluate the lipid profile (total cholesterol, LDL-C, triglycerides, and HDL-C) of stroke patients three months before they developed stroke in an attempt to understand the role of dyslipidemia in the occurrence of stroke. In addition to finding the differences between lipid profiles among hemorrhagic stroke and ischemic stroke patients.

## Materials and methods

This study was a retrospective time-series analysis of stroke patients who presented to the emergency department and was confirmed to have ischemic or hemorrhagic strokes by computed tomography (CT) from January 1, 2018, to December 31, 2020. This study was based on data extracted from an electronic hospital information system (BESTCare) of patients presenting to King Abdulaziz Medical City, National Guard Health Affairs, which is a tertiary academic medical center in Riyadh, Saudi Arabia. All male and female patients who were 14 years old and above, as per the hospital policy, were included in the study. Patients with coagulopathy diseases, who lack a lipid profile reading within six months before the incident, or who had a traumatic hemorrhagic stroke were excluded from the study. All these variables were included in the study: age, gender, height, weight, date of the incident, date of the last lipid profile results, type of stroke, comorbidities, on a statin or not, and lipid profile, including (HDL-C, LDL-C, total cholesterol, and triglycerides).

Ethical approval was granted from King Abdullah International Medical Research Centre. Consent was not required as it is a retrospective study and we used only aggregated data in this study, not any individualized data.

Microsoft Excel 2019 (Microsoft Corporation, Redmond, WA) was used for data entry and data cleaning, and the Statistical Package for the Social Sciences (SPSS) version 23 (IBM Corp., Armonk, NY) was used for data analysis and visualization of the results. Frequencies and percentages were generated for categorical variables while mean and standard deviation were calculated for quantitative variables. A test with a P-value of < 0.05 was considered significant.

## Results

The mean age of presentation of stroke was 68±13; 59% of patients were males and 41% were females. BMI ranged from 30±8. Obesity (BMI 30 or above) was predominant in both stroke subtypes, 51 and 43 for ischemic and hemorrhagic stroke, respectively. Among all comorbidities listed in Table 1, hypertension was the most predominant while 81% of patients have high blood pressure. Diabetes was present in 71% of the population. Of the participants in this study, 114 had ischemic stroke and 87 had a hemorrhagic type.

**Table 1:** Demographic characteristics of participants

Variable	Category	N	%
Gender	Male	118	59
	Female	83	41
Age (mean±SD)		68±13	
Body mass index (mean±SD)		30±8	
Hypertension	No	38	19
	Yes	163	81
Diabetes	No	61	30
	Yes	140	70
Dyslipidemia	No	182	92
	Yes	16	8
Atrial fibrillation	No	189	94
	Yes	12	6
Cardiac disease	No	178	89
	Yes	23	11
Respiratory disease	No	189	94
	Yes	12	6
Obesity	Yes	94	48
	No	104	53
Others	No	115	57
	Yes	86	43
On statin	No	76	38
	Yes	125	62
Type of stroke	Ischemic	115	57
	Hemorrhagic	86	43

The fasting serum lipid profile of 114 ischemic stroke patients showed raised serum total cholesterol in 23.7% of patients with mean serum total cholesterol of 4.3 mmol/L. Abnormal triglyceride was present in 32 patients with a mean of 1.38 ±0.78. Similarly, serum LDL was raised in 27 patients with a mean value of 2.65 ±1.27 mmol/L. HDL was below the normal reference range in 96.5% of ischemic stroke patients with a mean value of 1.02 ±0.27 mmol/L. For those 86 patients with hemorrhagic stroke, abnormal total cholesterol levels and abnormal LDL levels were present in 17 patients with a mean value of 4.05 ±1.08 mmol/L and 2.48 ±0.93 mmol/L, respectively. Serum triglyceride was increased in 28.6% of patients with a mean value of 1.43 ±0.73 mmol/L. Abnormal HDL was present in 83 patients with a mean value of 1.01 ±0.24 mmol/L. A comparison of the serum lipid profile of two categories of strokes showed no statistical significance in comparing serum values of total cholesterol, triglycerides, LDL-C, and in ischemic and hemorrhagic stroke patients (Tables 2-3). The ratios of TC/HDL, TG/HDL, and LDL/HDL were calculated, and all readings of both ischemic and hemorrhagic strokes were optimal (Table 4). For ischemic patients, TC/HDL, TG/HDL, and LDL/HDL presented as 4.2, 1.3, and 2.6, respectively (Table 5).

**Table 2:** Comparing ischemic and hemorrhagic strokes by baseline characteristics of participants

Variable	Category	Ischemic stroke		Hemorrhagic stroke		P-value
		N	%	N	%	
Gender	Male	68	59	50	58	0.852
	Female	47	41	36	42	
Age (mean±SD)		69	13	68	12	0.059
Body mass index (mean±SD)		30	18	29	8	0.682
Obesity	Yes	59	51	38	43	0.264
	No	56	49	48	57	
Hypertension	No	23	20	15	17	0.852
	Yes	92	80	71	83	
Diabetes	No	33	29	28	33	0.66
	Yes	82	71	58	67	
Atrial fibrillation	No	108	94	81	94	0.834
	Yes	7	6	5	6	
Cardiac disease	No	102	89	76	88	0.913
	Yes	13	11	10	12	
Respiratory disease	No	110	96	79	92	0.25
	Yes	5	4	7	8	
Other diseases	No	67	58	48	56	0.573
	Yes	48	42	38	44	
On statin	No	37	32	39	45	0.046
	Yes	78	68	47	55	

**Table 3:** Comparison of mean values of lipids in hemorrhagic and ischemic stroke

Type of lipid	Ischemic stroke		Hemorrhagic stroke		P-value
	Mean	SD	Mean	SD	
Total cholesterol	4.32	1.27	4.05	1.08	0.115
LDL cholesterol	2.65	1.02	2.48	0.93	0.227
HDL cholesterol	1.02	0.27	1.01	0.24	0.762
Triglycerides	1.38	0.78	1.43	0.73	0.677

**Table 4:** Comparison of abnormal lipid profiles in hemorrhagic and ischemic stroke participants

Type of lipid		Ischemic stroke		Hemorrhagic stroke		P-value
		N	%	N	%	
Abnormal cholesterol	No	87	76.3	67	79.8	0.564
	Yes	27	23.7	17	20.2	
Abnormal LDL	No	87	76.3	67	79.8	0.564
	Yes	27	23.7	17	20.2	
Abnormal HDL	No	4	3.5	1	1.2	0.304
	Yes	110	96.5	83	98.8	
Abnormal triglyceride	No	82	71.9	60	71.4	0.938
	Yes	32	28.1	24	28.6	

**Table 5:** Ratio between different lipoproteins

TC/HDL	4.2	4.0
TG/HDL	1.3	1.4
LDL/HDL	2.6	2.5

## Discussion

Our study provided a comprehensive analysis of stroke patients’ lipid profiles three months before they had a stroke in an attempt to understand the role of hyperlipidemia on the development of different stroke subtypes, namely, ischemic and hemorrhagic.

Age was recognized as an unmodifiable risk factor for stroke. And as the population of old people is expected to increase over the coming decades due to higher life expectancy, the stroke rate will increase in Saudi Arabia and globally [8-9]. According to a recent report by Aljadid et al. [10], stroke rates in Saudi Arabia were found to be higher in the 61-70 age group as compared to the 20-30 and 31-40 age groups. Our study findings were in agreement with the results from previous studies showing that the prevalence of stroke increases with age [1-4]. The age range in this study was from the 55-81 age group. Moreover, age influences the severity of the stroke and the functional ability of patients [10].

Multiple studies around the Kingdom found that hypertension and diabetes are major risk factors for stroke. A study done in Gizan found that 45% of stroke patients had hypertension and 28% had diabetes [11]. Another study done in the Asser region found that hypertension and diabetes were signiﬁcantly highly prevalent among stroke Saudi patients at 57.7% and 49.4%, respectively [4]. In the present study, we found that 80% of stroke patients had hypertension.

Diabetes mellitus was the second reported risk factor for stroke, present in 71% of the patients. According to WHO, Saudi Arabia is the second-highest country in the Middle East and the seventh globally for diabetes [11]. A tenfold increase in the prevalence of diabetes was noticed in Saudi Arabia over the past 30 years [11-12]. The health burden of diabetes and hypertension in Saudi Arabia is expected to increase to a serious level due to stroke. A recent study by Alhazzani revealed that people with diabetes have signiﬁcantly more than two times the chance to have a stroke compared to non-diabetic patients [11]. According to a recent study, the awareness of hypertension and diabetes as risk factors for stroke among the general population of Saudi Arabia is low [13]. Therefore, an effective preventive program against hypertension and diabetes is warranted. Through such a program, primary and secondary preventive measures will minimize the health impacts of diabetes, hypertension, and stroke eventually. According to a local study, 49.6% of the Saudi general population is obese [12]. In the present study, we found that 48% of stroke patients were obese (BMI above 30). A recent systematic review showed that obesity is an independent risk factor for stroke in the Middle East countries and is found in 66.0% of stroke patients [13]. Dyslipidemia has been identified as a risk factor for many diseases including hypertension, cardiac disease, and stroke [1]. Unfortunately, dyslipidemia is a high prevalence disease among the Saudi population. According to a recent local study, 32.1% of the general population of Saudi Arabia has dyslipidemia. In a recent systematic review, dyslipidemia was found in 65.8% of stroke patients in the Middle East countries [3].

The present study showed no significant difference between hemorrhagic and ischemic stroke in association with total cholesterol level. However, 23.7% of ischemic stroke patients and 20.2% of the hemorrhagic stroke group had elevated total cholesterol levels. Previous studies showed a significant relation between serum total cholesterol and ischemic stroke with an inverse relation with the hemorrhagic subtype [14].

The Copenhagen City Heart Study found a significant association between serum triglyceride and ischemic stroke [14]. Another prospective study over 13 years of follow-up found a significant association between increased levels of triglycerides and ischemic stroke in the general population [7]. High plasma triglyceride concentration was not significantly different for both types of stroke in this study, and only 28% of our sample had abnormal triglyceride levels. A similar finding was found in another study conducted in India, they found that triglycerides have no association with either stroke type [15].

Many randomized control trials demonstrated LDL as an important causal risk for the development of Ischemic stroke and reported an inverse association of LDL with the risk of hemorrhagic stroke [16]. Our study observed the null associations difference of LDL in hemorrhagic and ischemic stroke patients. 

HDL plays an essential role as anti-atherogenic lipid by which excess cholesterol is removed from the peripheral vessels and is transported back to the liver [14]. According to the Northern Manhattan Study (NOMAS), a higher HDL level was protective against ischemic stroke in all racial groups, especially in those 75 years or older [17]. Among all lipoproteins, HDL was the least type of lipid to be optimal in our sample of Saudi patients, only 3.5% and 1.2% of ischemic and hemorrhagic stroke patients had optimal HDL, respectively. 

We found a significant difference in the use of statin between ischemic and hemorrhagic stroke patients, 68% and 54%, respectively. This was statically significant with a p-value of 0.04. Several clinical trials have found that statins reduced the risk of ischemic stroke and other vascular diseases, but it increases the risk of hemorrhagic stroke [1]. Patients are more likely to be prescribed statins in an attempt to reduce their risk of cerebrovascular disease [17], and this may, in part, explain our findings.

Our study has several strengths, including the role of dyslipidemia control in the development of stroke which is still an active area for research with different controversial results. Analysis of different lipoproteins levels may help future research.

Due to the lack of local studies comparing lipid profiles of ischemic and hemorrhagic stroke among Saudi patients, our study may help further analytic case-control studies. This study has some limitations that include being in one region of Saudi Arabia, and only the patients admitted to one hospital were included. Furthermore, as our study was a retrospective one, the compliance of patients prescribed statin before they developed stroke was not assessed, which might affect their lipid profile result. Further national-based studies are needed.

## Conclusions

Most acute stroke patients have hypertension and diabetes. Not being on a statin for dyslipidemia patients increases the risk of having hemorrhagic strokes more than ischemic strokes. A lipid profile before the stroke doesn't predict the type of stroke.
